# Persistent Ketamine-Induced Cholangiopathy: An Approach to Management

**DOI:** 10.7759/cureus.11611

**Published:** 2020-11-21

**Authors:** Tiwonge J Nyirenda, Ahmad Shirazi-Nejad, Ashraf S Soliman

**Affiliations:** 1 Gastroenterology, Barnsley Hospital NHS Foundation Trust, Barnsley, GBR

**Keywords:** ketamine, hyperbilirubinaemia, cholangitis, biliary stent, liver transplant

## Abstract

A 32-year-old man presented with profound jaundice, rigors and decreased appetite. Initial liver function tests (LFTs) were deranged in a cholestatic pattern with imaging demonstrating a dilated biliary system, with no filling defects. It has been observed that LFTs typically improve upon ketamine cessation, but this case demonstrated escalating hyperbilirubinaemia, despite ketamine cessation. Recurrent cholangitis and biliary duct stricturing were demonstrated on magnetic resonance cholangiopancreatography (MRCP). This prompted investigation of other biliary pathology and consideration for intervention.

## Introduction

Ketamine is widely used for its anaesthetic properties in surgical procedures, as a non-competitive inhibitor of the N-methyl-D-aspartate (NMDA) receptor in the brain. When used illicitly, the powder can be taken orally or inhaled to cause dissociative phenomena and hallucinations [1]. We report a case of chronic ketamine abuse causing recurrent cholangiopathy requiring endoscopic biliary stenting. There is no current consensus document or guideline on the approach to the management of persistent biliary dilatation and cholangitis, after ketamine cessation.

## Case presentation

A 32-year-old man presented to the emergency department with jaundice, rigors and decreased appetite for a week. Initial clinical examination revealed jaundice, right hypochondrium pain and tender hepatomegaly. He was alert with a Glasgow Coma Scale (GCS) 15/15 but a temperature 38.9°C. He also reported urinary frequency and urgency.

Review of his medical records revealed a previous admission a month ago, with deranged liver function tests (LFTs). He had a bilirubin (bili) 18µmol/L (<20µmol/L), alanine aminotransferase (ALT) 203U/L (<40U/L), aspartate aminotransferase (AST) 86U/L (<40U/L), alkaline phosphatase (ALP) 1701U/L (30-130U/L), gamma glutamyl transpeptidase (GGT) 2050U/L (<50U/L). Magnetic resonance cholangiopancreatography (MRCP) showed dilated bile ducts with no filling defects, gallstones or strictures. LFTs had spontaneously resolved without medical intervention and no cause identified for the dilated bile ducts. Discharge LFTs were: bili 13µmol/L, ALT 63U/L, AST 66U/L, ALP 1335U/L, and GGT 1554U/L.

Admission biochemical indices revealed: white cell count (WCC) 25x109/L (3.6-11x109/L) and C-reactive protein (CRP) 317mg/L (<5mg/L), bili 196µmol/L, ALT 184U/L, AST 227U/L, ALP 2151U/L, GGT 2282U/L, creatinine 310µmol/L (60-120µmol/L), and estimated glomerular filtration rate (eGFR) 22mL/min/1.73m2. He was empirically treated for biliary sepsis but within 24 hours became drowsy with a GCS of 10/15. Peripheral neurological examination was normal with a normal plantar reflex. Encephalopathy was presumed, but there was no improvement with a lactulose enema. Computed tomography (CT) head scan, ultrasound scan (USS) abdomen and urinary drug screen were unremarkable.

The otolaryngologists removed a large ketamine crystal from the left nostril however his GCS continued to deteriorate, which required admission to the intensive care unit (ICU). It transpired that for the past 15 years, he had been nasally inhaling ketamine daily. One week into his inpatient stay, the LFTs continued to deteriorate: bili 329µmol/L, ALT 113U/L, AST 77U/L, ALP 2917U/L, GGT 2319U/L. The non-invasive liver screen (NILS) including c-antineutrophil cytoplasmic antibodies (ANCA) and p-ANCA, all came back negative. Repeat MRCP showed diffuse mural irregularity of the intra and extrahepatic bile ducts typical for diffuse cholangitis, this fitted with his initial presentation of symptoms. His bilirubin slowly improved down to 57µmol/l with a creatinine 165µmol/L at discharge.

In outpatient clinic, he was diagnosed with recurrent cholangitis. Despite treatment in the community, repeat bloods revealed: bili 249µmol/L, WCC 24x109/L, CRP 135mg/L, creatinine 226µmol/L. His renal function deteriorated and venous blood gas (VBG) showed metabolic acidosis. USS showed no change of known bilateral hydronephrosis, compared to his initial presentation USS, two months ago. The nephrologists recommended sodium bicarbonate administration. He denied resuming ketamine consumption but still had symptoms of urinary frequency and urgency. The nephrologists felt that his renal function was unlikely to improve until he is stable enough to undergo bladder reconstruction surgery for ketamine bladder syndrome.

A third MRCP revealed biliary duct stricturing, more predominant and extensive in intrahepatic ducts with features of cholangitis. This raised the concern of ongoing cholangiopathy related to ketamine and recurrent biliary sepsis, but also the possibility of a secondary sclerosing cholangitis. A liver biopsy was not deemed necessary given his pristine NILS screen that were taken twice. This included a negative autoimmune liver profile both times too. With negative results for human immunodeficiency viruses, alfa-fetoprotein and immunoglobulins, the overwhelming evidence all pointed towards a diagnosis of ketamine-induced cholangiopathy with recurrent infective cholangitis.

Endoscopic Retrograde Cholangio-Pancreatography (ERCP) was performed and two straight plastic stents were placed in the hilar stricture. There was a concordant reduction in bili to 178µmol/L. He was referred to the transplant assessment clinic due to recurrent episodes of cholangitis and chronically elevated bilirubin, despite optimum medical therapy over a six-month period.

**Figure 1:**
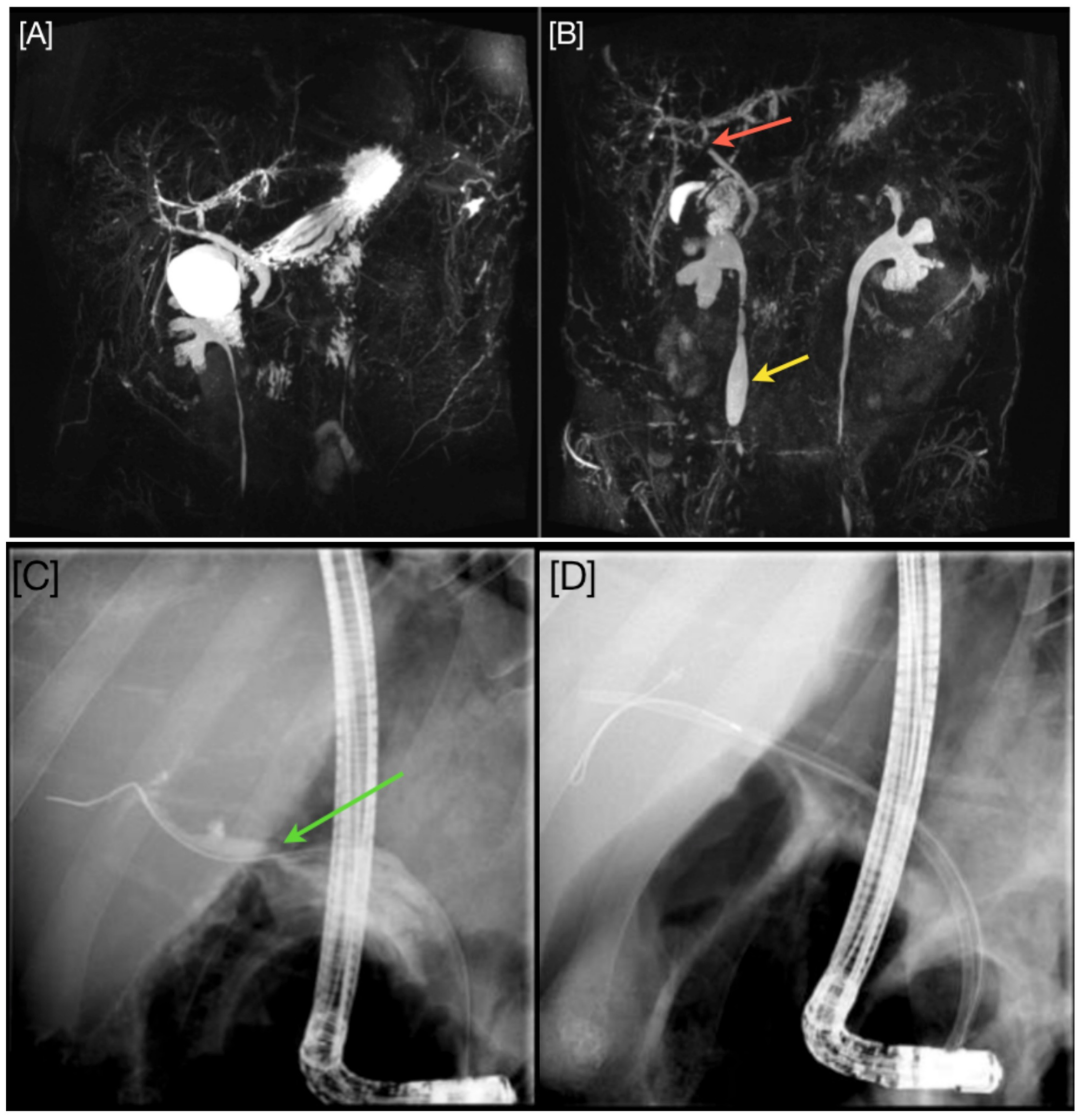
[A], Magnetic resonance cholangiopancreatography (MRCP) before initial presentation on the left compared to [B], the updated MRCP on the right after 4 months. [A] displays a dilated biliary system with no filling defects while [B] shows biliary ducts stricturing with a prominent stricture at the hilar (red arrow). Note the moderate right sided hydronephrosis (yellow arrow). [C], Endoscopic Retrograde Cholangio-Pancreatography (ERCP) with guide wire through the hilar stricture (green arrow) and [D] ERCP, showing placement of the second straight plastic stent.

## Discussion

Short-term use of ketamine has been associated with elevated transaminases, resembling a drug-induced liver injury (DILI) pattern [1]. Chronic use is associated with abnormalities in the biliary system of unknown mechanism [1,2]. Lui et al. suggest ketamine causes NMDA receptor stimulation within smooth muscle cells of the bile duct leading to inflammation, fibrosis and stricturing [2]. In their case, a biopsy was performed that displayed mild to moderate portal fibrosis with ductular proliferation, which was also seen in Haghi et al.’s case [2,3].

Ketamine is metabolised by the cytochrome P450 system in the liver before being excreted in bile and urine after glucuronoconjugation [4]. Therefore, it has also been hypothesised that pathology could be due to direct toxic injury to the surface epithelium [1]. Al-Nowfal proposed that biliary dilatation may be due to increased resistance to flow across the sphincter of Oddi [4]. Wong et al.’s case series showed improvement in common bile duct (CBD) diameter and LFTs, after biliary stent placement, regardless of whether there was a radiologically confirmed obstructive lesion [5].

Considering previous case reports, ketamine possibly exerts its effects on the biliary system via direct toxic injury to biliary epithelial cells, chronic inflammation leading to fibrosis and impairment of the sphincter of Oddi [4]. This results in a form of sclerosing cholangitis with biliary duct dilatation. Cessation of ketamine use allows for slow improvement of deranged LFTs and reversal of the abnormalities seen on imaging several months later [1]. However our case demonstrates evidence of progression in bile duct dilatation and strictures on subsequent MRCPs over a six-month period. 

## Conclusions

Ketamine abuse should be included in the differential diagnosis when investigating deranged LFTs with worsening renal function. Our case has shown the development of a sclerosing cholangitis picture secondary to chronic ketamine abuse with relentless progression despite abstinence. The main treatment is cessation of ketamine, though if a chronic stricture develops, biliary stenting may be necessary to relieve any obstruction identified on MRCP. The nature of all the cases discussed displays how a critical review of medication history, especially recreational drugs, is important.
